# Regulation of neural stem cell proliferation and differentiation by Kinesin family member 2a

**DOI:** 10.1371/journal.pone.0179047

**Published:** 2017-06-07

**Authors:** Dong Sun, Xue Zhou, Hua-Li Yu, Xiao-Xiao He, Wei-Xiang Guo, Wen-Cheng Xiong, Xiao-Juan Zhu

**Affiliations:** 1 Key Laboratory of Molecular Epigenetics of Ministry of Education, Institute of Cytology and Genetics, Northeast Normal University, Changchun, Jilin, China; 2 Department of Neuroscience & Regenerative Medicine and Department of Neurology, Augusta University, Augusta, Georgia, United States of America; 3 State Key Laboratory for Molecular and Developmental Biology, Institute of Genetics and Developmental Biology, Chinese Academy of Sciences, Beijing, China; Lewis Katz School of Medicine at Temple University, UNITED STATES

## Abstract

In the developing neocortex, cells in the ventricular/subventricular zone are largely multipotent neural stem cells and neural progenitor cells. These cells undergo self-renewal at the early stage of embryonic development to amplify the progenitor pool and subsequently differentiate into neurons. It is thus of considerable interest to investigate mechanisms controlling the switch from neural stem cells or neural progenitor cells to neurons. Here, we present evidence that Kif2a, a member of the Kinesin-13 family, plays a role in regulating the proliferation and differentiation of neural stem cells or neural progenitor cells at embryonic day 13.5. Silencing Kif2a by use of in utero electroporation of Kif2a shRNA reduced neural stem cells proliferation or self-renewal but increased neuronal differentiation. We further found that knockdown of Kif2a decreased the protein level of β-catenin, which is a critical molecule for neocortical neurogenesis. Together, these results reveal an important function of Kif2a in embryonic neocortical neurogenesis.

## Introduction

Embryonic brain development is a complex process that includes the proliferation and differentiation of neural stem cells (NSCs) and neural progenitor cells (NPCs), neuronal migration and polarity establishment [1–4]. In the ventricular zone/subventricular zone (VZ/SVZ), NSCs/NPCs originated from neuroepithelial cells continuously proliferate and produce new neurons to form a six-layered laminar structure, which is necessary for proper brain functions [5,6]. During the peak stage of embryonic neurogenesis, NSCs/NPCs from embryonic day 13.5 (E13.5) mainly undergo asymmetric divisions to self-renewal and generate one neuron or one intermediate progenitor cell (IPC), which subsequently divides symmetrically in the SVZ and differentiates into two neurons [7]. Several signaling pathways are known to be involved in neocortical neurogenesis: for example, the canonical Wnt signaling pathway (Wnt-β-catenin pathway) has been well studied, and its role in the mechanisms of neurogenesis is clearly understood. Wnt-β-catenin signaling has been reported to be activated in radial glia cells (RGCs) and IPCs and then down-regulated in differentiating cells as they migrated away from the VZ [8,9]. The balance between NSCs/NPCs proliferation/self-renewal and differentiation ensures an appropriate number of neurons are generated, which is a critical step for neocortical neurogenesis [10]. Therefore, the mechanisms underlying neurogenesis during embryonic development have received considerable interest.

Kinesin family member 2a (Kif2a), a member of the Kinesin-13 family, was first identified as a kinesin motor protein that binds at the plus end of microtubules (MTs) and depolymerizes MTs depending on the activity of adenosine triphosphate (ATP). MTs are a major component of the cytoskeleton, which are composed of α-tubulin, β-tubulin and other associated proteins and exhibit a dynamic process of depolymerization and aggregation [11,12]. MTs are involved in various cellular processes including cell division, migration, polarity and morphology [13–15]. As Kif2a binds to the end of MT, it will take advantage of the energy of ATP hydrolysis, and subsequently, α-tubulin and β-tubulin will dissociate from MTs [16], indicating that Kif2a is not involved in transporting cargos such as membranous organelles and macromolecules [17]. As is reported, Kif2a knockout (*Kif2a*^*-/-*^) mice showed multiple abnormalities, such as died within one day of birth, ventricle enlargement, lamination defects, and lack of nerve nuclei in normal localizations [18]. In the developing neurons, Kif2a regulated MT dynamics at the growth cone edge, and *Kif2a*^*-/-*^ neurons displayed more elongated collateral branches than those in the wild-type mice [18,19]. Kif2a mutation in humans has also been reported to result in lissencephaly, developmental delay, and infantile spasms [20,21]. Besides functioning in brain development, Kif2a also plays important roles in other aspects, such as regulating cell migration [22], controlling primary cilia disassembly [23], building a bipolar spindle and regulating chromosome segregation [24,25], and tumorigenesis of several types of tumors [26]. However, the function of Kif2a in embryonic NSCs/NPCs as well as in neocortical neurogenesis remains poorly understood.

In our study, we found that Kif2a played an important role in neocortical neurogenesis. Knocking down Kif2a inhibited NSCs/NPCs proliferation but promoted neuronal differentiation in vivo and in vitro. In the developing embryos, newborn cells derived from E13.5 Kif2a-deficient NSCs/NPCs were abnormally distributed. More newborn cells were located in the intermediate zone (IZ) and cortical plate (CP), and fewer newborn cells resided in the VZ/SVZ at E15.5 in Kif2a shRNA-expressing brains than those in control brains, supporting a model of accelerating neuronal differentiation. Meanwhile, we found that β-catenin protein level was decreased after knocking down Kif2a. Furthermore, the phosphorylation of AKT and GSK3β was also decreased in Kif2a-deficient cells, which may partially explain the role of Kif2a in the proliferation and differentiation of NSCs/NPCs. Thus, these results suggest that Kif2a plays a critical role in promoting NSCs/NPCs proliferation or self-renewal and suppressing neuronal differentiation, thereby maintaining brain cell homeostasis.

## Materials and methods

### Animals

Mice of the C57BL/6 background were handled according to the Guidelines for the Care and Use of Laboratory Animals. The project was approved by the Institution Animal Care and Use Committee of Northeast Normal University. All mice were housed in ventilated cages, given food and water and maintained with a 12-h light/dark cycle. During in utero electroporation, pregant mice were anesthetized by Pelltobarbitalum Natricum (70mg/kg). After wake up, the mice were allowed to maintain for expected time. To get brains, 10 minutes of exposure to Carbon dioxide (CO_2_) was used for euthanasia of mice.

### Antibodies

Primary antibodies used included rabbit anti-Kif2a (1: 1000, Abcam), rabbit anti-Tbr2 (1:500, Abcam), rabbit anti-Pax6 (1:200, Covance), mouse anti-beta III tubulin (TU20) (1:500, Abcam), mouse anti-nestin (1:500, Abcam), mouse anti-α tubulin (1:5000, CST), rat anti-Brdu (1:500, Abcam), rabbit and chicken anti-green fluorescent protein (1:500, GFP) (Invitrogen and Aves, respectively), rabbit anti-Ki67 (1:500, Thermo), rabbit anti-PH3 (1:300, Millipore), rabbit anti-cleaved caspase3 (1:300, Cell signaling), rabbit anti-AKT (1:500, CST), rabbit anti-p-AKT (1:500, CST), rabbit anti-β-catenin (1:200, Santa Cruz), mouse anti-GSK3β (1:200, Santa Cruz), and mouse anti-p-GSK3β (1:200, Santa Cruz). All the corresponding conjugated secondary antibodies were purchased from Invitrogen. Nuclei were stained with 4′,6-diamidino-2-phenylindole (DAPI) (Roche).

### Plasmids

Kif2a shRNAs were generated by inserting hairpin oligonucleotides into the pSUPER vector plasmid, and the target sequences used were GCTGAAGAAGCCAAACTAT (shRNA1) and GGAATGGCATCCTGTGAAA (shRNA2). The scramble shRNA was used as a negative control which contained no homology to the known mammalian genes. The Kif2a shRNA1-Resistant (Kif2a^Res^) plasmid expressed Kif2a but was not sensitive to Kif2a shRNA1.

### Western blot

NLT cells transfected with scramble shRNA or Kif2a shRNAs were lysed in cell lysis buffer containing 20 mM Tris-HCl (pH 7.4), 150 mM NaCl, 1% NP-40, 0.5% Triton X-100, 1 mM phenylmethylsulfonyl fluoride (PMSF), 1 mM EDTA, 5 mM sodium fluoride, and 2 mM sodium orthovanadate and supplemented with a protease inhibitor cocktail (Roche). Samples were incubated with cell lysis buffer for 20 minutes on ice and oscillated every 5 minutes and then centrifuged at 12,000 rcf for 15 minutes. Based on the protein weight, we used 10% SDS-PAGE gels to separate proteins and then transferred them onto the nitrocellulose (NC) membrane. After electrotransfer, nitrocellulose membranes were blocked in Western wash solution plus 5% low fat dried milk for 1 hour at room temperature. Relative primary antibodies were diluted in proper concentrations to incubate NC membrane overnight at 4°C. Membranes were washed and incubated for 1 h at room temperature with an appropriate horseradish-peroxidase-conjugated secondary antibody (1:5,000, Thermo). For visualization of the signal, we used an ECL kit (Pierce, Rockford, IL). For quantitative analysis, protein bands detected by ECL were scanned and then analyzed using ImageJ software.

### In utero electroporation

In utero electroporation experiments were performed as described previously with slight modification [3]. At E13.5, pregnant mouse was anesthetized with Pelltobarbitalum Natricum (70mg/kg), and embryos were exposed on humidified gauze pads. About 2 μl of DNA solutions (2 μg/μl) plus 0.01% Fast Green (Fluka) were injected into the lateral ventricles of the embryonic brain with a glass micropipette (GD-1, 1mm outer diameter × 90mm length, NARISHIGE). For electroporation, 5×50 ms, 29 V square pulses separated by 950 ms intervals were delivered with forceps-type electrodes connected to an ECM 830 electroporator (BTX Harvard Apparatus). The uterus was then carefully replaced into the abdominal cavity, and the wound was sutured using the surgical needle and thread. The pregnant mouse was warmed in an incubator until it became conscious, and embryos were allowed to develop in utero for the indicated time.

### Brdu labeling

For in vivo experiments, 5-bromodeoxyuridine (Brdu) was administered to E15.5 mice via a single injection (50 mg/kg) 2 hours before sacrifice. The brains were collected from embryos and processed for immunohistochemistry as described below. For in vitro experiments, we cultured NSCs from E13.5 cortex and plated them onto poly-L-ornithine and laminin-coated coverslips at a cell density of 1×10^5^ cells/ml. After 24 hours, we mixed Brdu (5 μM) into culture medium (Neurobasal medium+B27+GlutaMAX+20ng/ml of FGF-2+20ng/ml of EGF) and cultured cells for 6 hours. The coverslips were then collected for cell proliferation analysis via immunofluorescent staining.

### Tissue collection and immunostaining

Embryonic brains of electroporation were separated in phosphate buffered saline (PBS) (pH 7.4), and then fixed in 4% (wt/vol) paraformaldehyde (PFA) dissolved in PBS overnight at 4°C. After being washed with PBS, brains were immersed in 30% (wt/vol) sucrose solution dissolved in PBS, and we renewed the sucrose solution every day for complete dehydration. After approximately 4 days, brains were embedded in 1 cm×1 cm boxes with optimal cutting temperature compound (O.C.T.) compound (Tissue-Tek), which is an embedding medium for frozen tissue specimens to ensure optimal cutting temperature, then cut into 20 μm sections using a freezing microtome (Leica).

For immunohistochemistry, the sections were incubated in PBS buffer for 5 minutes, and then antigens were unmasked by incubation in citrate buffer (pH 6.0) for 5 minutes at 96°C. After being returned to room temperature, the sections were washed 3 times (5 minutes each) and incubated overnight at 4°C with the primary antibody diluted in PBS containing 0.2% Triton X-100 and 2% bovine serum albumin (BSA). After being washed 3 times, sections were incubated with the corresponding conjugated secondary antibody for 1.5 hours. Nuclei were stained using DAPI.

For immunofluorescent staining, the coverslips were washed 3 times (5 minutes/time) with PBS and then fixed with 4% PFA dissolved in PBS for 20 minutes at room temperature. After being washed 3 times, they were incubated overnight at 4°C with primary antibody in PBS containing 0.1% Triton X-100 and 2% BSA. The next day, the cells were washed with PBST (PBS with 0.1% Tween-20) and incubated with secondary antibody for 1 hour. Nuclei were counterstained using DAPI.

### Production of lentivirus

Scramble shRNA or Kif2a shRNA (the same target sequence as Kif2a shRNA1) mixed with the packaging plasmids PAX2 and PMD2G were transfected into HEK 293 cells using polyethyleneimine (PEI). We then collected supernatant at 48, 72 and 96 hours post-transfection, filtered it using a 0.2 μm filter, and then concentrated it at 20,000 rcf for 2 hours at 4°C. Finally, we removed the supernatant and resuspended the lentivirus in 100 μl of PBS.

### Cell lines

The normal gonadotropin-releasing hormone (GnRH) neuronal cell line transfected with the large T antigen (NLT) was obtained from Dr. W.C. X (Department of Neuroscience & Regenerative Medicine and Department of Neurology, Augusta University, Augusta, GA 30912, USA).

Neurospheres were prepared and maintained as described previously with slight modifications [27,28]. Briefly, cerebral cortex was isolated from E13.5 embryos and then treated with 0.25% trypsin for 15 minutes at 37°C, and trypsin inhibitor was added to inactivate the trypsin. After centrifuged at 200 rcf for 5 minutes at room temperature, we resuspended cells and seeded them in a 10 cm plate with proliferation medium containing Neurobasal medium, B27 supplement, glutamine, human recombinant epidermal growth factor (hEGF) (20 ng/ml) and basic fibroblast growth factor (b-FGF) (20 ng/ml). After approximately 3 days, the neurospheres were passaged to produce secondary neurospheres.

For neuronal differentiation assays, NSCs were plated onto coverslips coated with poly-L-ornithine and laminin. We cultured NSCs overnight in a 37°C culture incubator and then replaced the proliferation medium with differentiation medium for another 3 days in culture. Finally, differentiated cells were collected for immunofluorescence staining with the indicated antibodies.

### Statistical analysis

All results presented in this study were obtained from at least three independent experiments. Statistical analyses were performed using Prism 5.0 (GraphPad Software). Two groups of data were compared using Student’s t test, and one-way ANOVA followed by Bonferroni post-hoc test was used to analyze 3 or more groups comparisons. All data are expressed as the mean ± SEM and described in the figure legends. A value of *P* < 0.05 was considered to be significant.

## Results

### Kif2a is expressed in NSCs/NPCs as well as neurons of the developing neocortex

**Fig 1:**
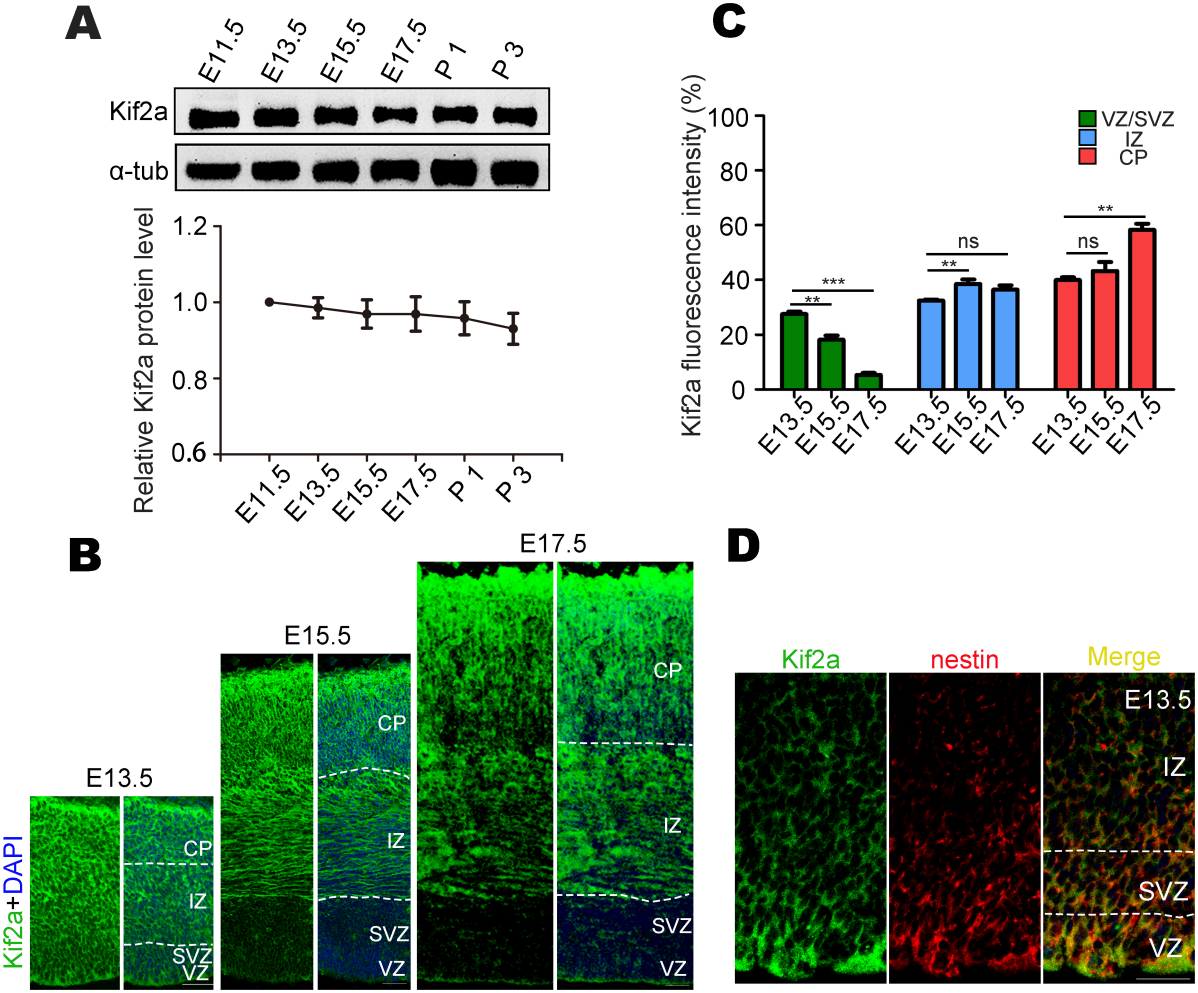
Expression and distribution of Kif2a in developing cortex. **(A)** Western blot and quantitative analysis of Kif2a protein levels at different developmental stages. **(B)** Immunostaining of E13.5, E15.5 and E17.5 brain slices. Kif2a (green) is expressed throughout the whole neocortex at E13.5; however, at E15.5 and E17.5, it is highly expressed in the IZ and CP and weakly detected in VZ/SVZ. IZ, intermediate zone; CP, cortical plate. Scale bars = 50 μm. **(C)** Quantitative analysis of the fluorescence intensity of Kif2a (green) immunostaining in different regions of neocortex. **P < 0.01, ***P < 0.001, ns = no significant difference; one-way ANOVA followed by Bonferroni post-hoc test. Data are presented as the mean ± SEM. **(D)** Kif2a (green) is co-expressed with nestin-positive NSCs/NPCs (red) in the VZ/SVZ at E13.5. Scale bars = 40 μm.

To investigate the potential roles of Kif2a in the embryonic neocortex, we examined its expression and distribution. Western blot analysis of neocortical homogenates showed that Kif2a was expressed at similar levels from E11.5 to postnatal day (P) 3 (Fig 1A). At the peak stage of neocortical neurogenesis (E13.5), immunostaining showed that Kif2a was expressed throughout the whole neocortex (Fig 1B, left). Interestingly, Kif2a was largely distributed in the IZ and CP but only weakly detected in the VZ/SVZ at E15.5 and E17.5 (Fig 1B, middle and right). To compare the relative fluorescence intensity of Kif2a in different layers, we measured brain slices using the same laser power. Quantitative analysis showed that the percentage of cells expressing Kif2a over the whole neocortex was gradually reduced in the VZ/SVZ from E13.5 to E17.5 but was significantly increased in the IZ at E15.5 and in the CP at E17.5 (Fig 1C), indicating that Kif2a may play multiple roles with spatially and temporally distinct localization. The distribution of Kif2a at E13.5 was further confirmed by co-immunostaining analysis of Kif2a with nestin, a well-characterized NSCs/NPCs marker that is presented in the VZ/SVZ of mouse brain (Fig 1D). These results suggested that Kif2a may play a role in the VZ/SVZ at E13.5, a crucial time window for the amplification of NSCs/NPCs and for the generation of neurons.

### Knockdown Kif2a impairs the neocortical localization of newborn cells

**Fig 2:**
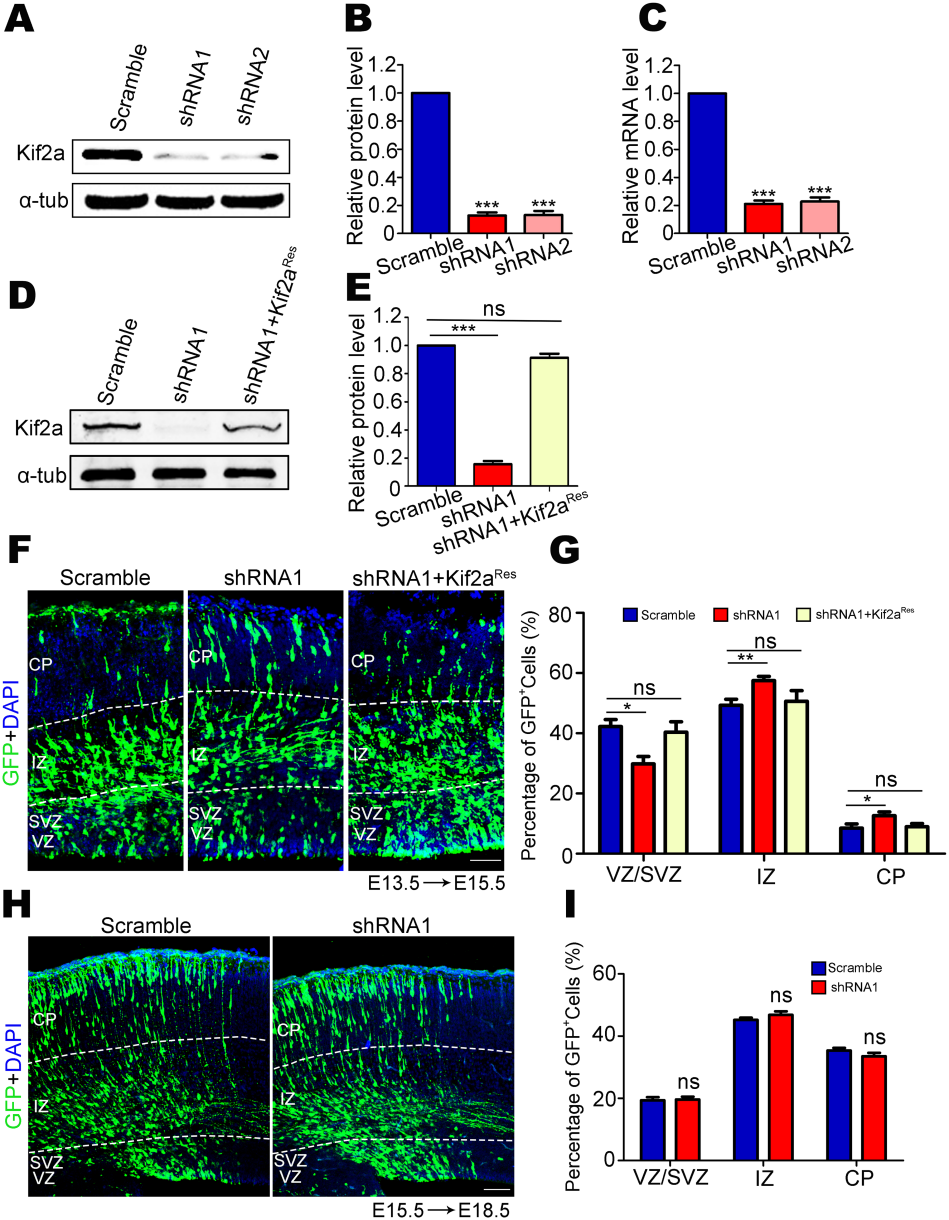
Knockdown Kif2a in E13.5 cortical NSCs/NPCs impaired the neocortical localization of newborn cells. **(A)** Western blot analysis of Kif2a expression in lysates of NLT cells transfected with scramble shRNA or Kif2a shRNAs. **(B)** Quantitative analysis of Western blot data in A. n = 3 per group. ***P < 0.001; one-way ANOVA followed by Bonferroni post-hoc test. Data are presented as the mean ± SEM. **(C)** Quantitative analysis of real-time PCR data for relative mRNA level in NLT cells transfected with scramble shRNA or Kif2a shRNAs. n = 3 per group. ***P < 0.001; one-way ANOVA followed by Bonferroni post-hoc test. Data are presented as the mean ± SEM. **(D)** Western blot analysis of Kif2a expression in lysates of NLT cells transfected with different plasmids (scramble shRNA, Kif2a shRNA1 or Kif2a shRNA1+Kif2a^Res^). **(E)** Quantitative analysis of Western blot data in D. n = 3 per group. ***P < 0.001; ns = no significant difference; one-way ANOVA followed by Bonferroni post-hoc test. Data are presented as the mean ± SEM. **(F)** Mouse embryos were electroporated with the indicated plasmids (scramble shRNA, Kif2a shRNA1 or Kif2a shRNA1+Kif2a^Res^) at E13.5 and analyzed at E15.5. GFP (green) represents cells expressing the indicated plasmids. Scale bars = 50 μm. **(G)** Quantitative analysis of the location of newborn cells in different regions of the neocortex. n = 800–1000 cells from three different brains. *P < 0.05, **P < 0.01, ns = no significant difference; one-way ANOVA followed by Bonferroni post-hoc test. Data are presented as the mean ± SEM. **(H)** Mouse embryos were electroporated with scramble shRNA or Kif2a shRNA1 at E15.5 and then analyzed at E18.5. GFP (green) represents cells expressing the indicated plasmids. Scale bars = 50 μm. **(I)** Quantitative analysis of the localization of newborn cells in different regions of neocortex. n = 600–800 cells from three different brains. ns = no significant difference; Student’s *t*-test. Data are presented as the mean ± SEM.

To address the role of Kif2a in neocortical neurogenesis, two shRNA constructs that silencing Kif2a expression were generated, and both shRNAs indeed efficiently knocked down endogenous expression of Kif2a in NLT cells (Fig 2A and 2B). The mRNA levels of Kif2a in NLT cells expressing Kif2a shRNAs were also significantly decreased compared to those in control cells (Fig 2C). In addition, expression of Kif2a^Res^ plasmids that resisted Kif2a shRNA1 restored Kif2a expression to a normal level (Fig 2D and 2E).

Scramble or Kif2a shRNA1 plasmids were in utero electroporated into E13.5 mouse brains, and the brain slices were examined at E15.5 (Fig 2F). Firstly, we investigated the distribution of GFP^+^ cells, representing newborn cells in the neocortex, by counting GFP^+^ cells in different layers. Knocking down Kif2a dramatically decreased the proportion of newborn cells that were present in the VZ/SVZ: among the total GFP^+^ cells, the percentage of GFP^+^ cells in the VZ/SVZ was significantly lower. However, the percentage of GFP-labeled cells in the IZ and CP was much higher in Kif2a shRNA1-expressing brains than that in control brains (Fig 2G), suggesting that Kif2a plays an important role in generation of cells in the SVZ. Furthermore, expression of Kif2a^Res^ plasmids rescued the phenotype of over-accumulation of newborn cells in the IZ and CP (Fig 2F and 2G).

We next investigated whether Kif2a has a similar function in E15.5 neocortical NSCs/NPCs. Thus, scramble shRNA or Kif2a shRNA1 was introduced into E15.5 NSCs/NPCs via in utero electroporation, and the localization of GFP^+^ cells in the neocortex was analyzed in E18.5 mouse brains (Fig 2H). Quantitative analysis showed that the percentage of GFP^+^ cells in each region was not significantly different between the two groups (Fig 2I), suggesting that Kif2a had little effect on neuronal migration. Taken together, Kif2a contributes to regulating the neocortical localization of newborn cells at E13.5.

### Kif2a regulates NSCs/NPCs’ proliferation in vivo and in vitro

**Fig 3:**
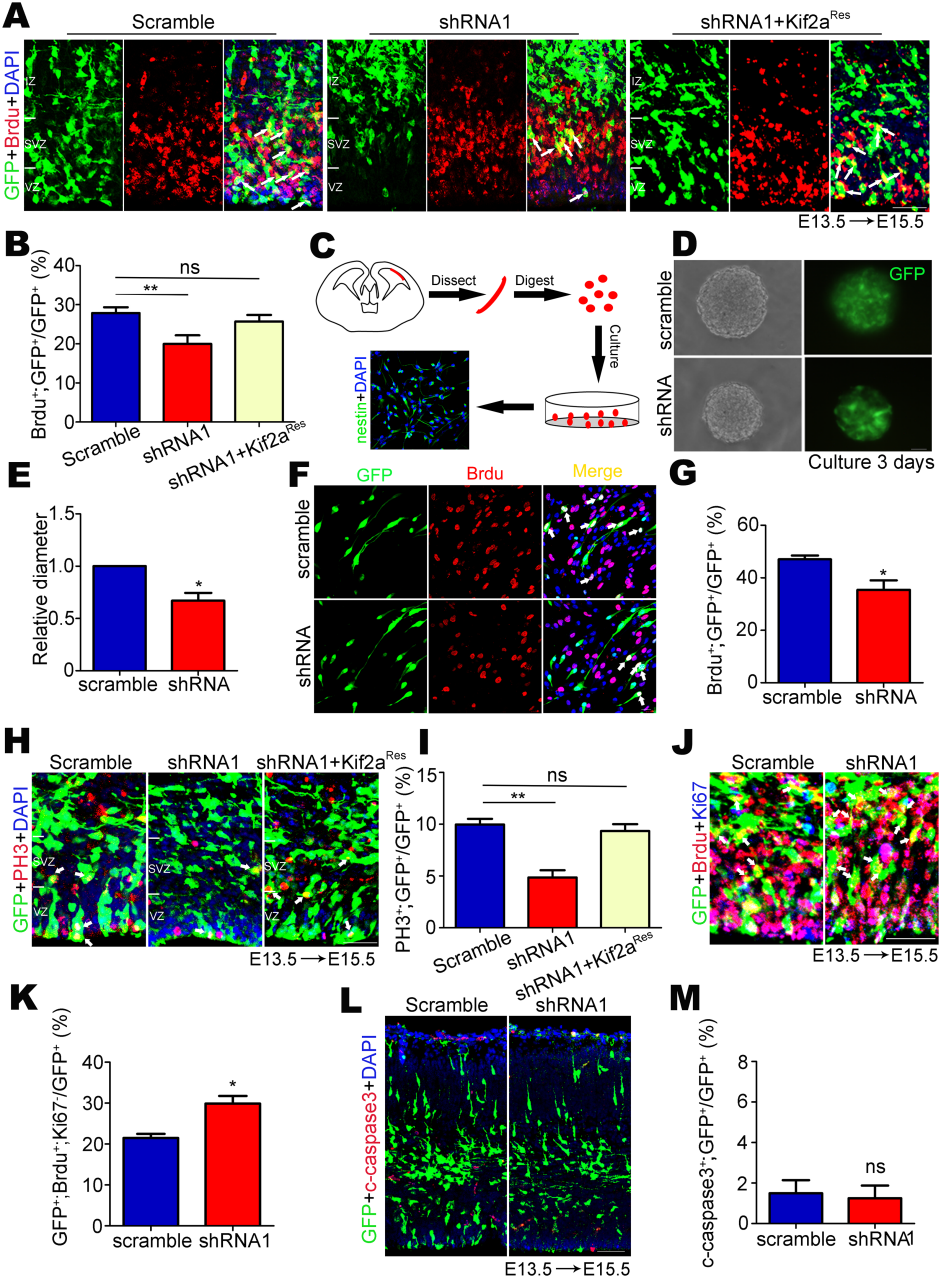
Kif2a regulated the proliferation or self-renewal of NSCs/NPCs in vivo and in vitro. **(A)** Indicated plasmids (scramble shRNA, Kif2a shRNA1 or Kif2a shRNA1+Kif2a^Res^) were introduced into E13.5 neocortex via in utero electroporation in vivo. Scale bars = 50 μm. **(B)** Quantitative analysis of the percentage of GFP^+^;Brdu^+^ cells among total GFP^+^ cells showed that Kif2a deficiency impaired the Brdu incorporation of NSCs/NPCs in vivo. n = 800–1000 cells from three different brains. **P < 0.01; ns = no significant difference; one-way ANOVA followed by Bonferroni post-hoc test. Data are presented as the mean ± SEM. **(C)** Schematic drawing showed the isolation of NSC/NPCs from E13.5 neocortex. **(D)** The NSCs/NPCs were transfected with lentivirus expressing scramble shRNA or Kif2a shRNA for 3 days. GFP (green) indicated the transfected cells. Scale bars = 50 μm. **(E)** Quantification of neurospheres size after transfection with scramble shRNA or Kif2a shRNA. n > 100 neurospheres from 3 different experiments. *P < 0.05; Student’s t-test. Data are presented as the mean ± SEM. **(F)** Cultured NSCs/NPCs were labeled with Brdu for 6 h. Scale bars = 10 μm. **(G)** Quantification of the percentage of GFP^+^;Brdu^+^ among total GFP^+^ cells showed that knocking down Kif2a inhibited the proliferation of NSCs/NPCs in vitro. n = 1300–1500 cells from 4 different experiments. *P < 0.05; Student’s *t*-test. Data are presented as the mean ± SEM. **(H)** Immunostaining of GFP (green) and PH3 (red) of E15.5 neocortex. Scale bars = 50 μm. **(I)** Quantification of the percentage of GFP^+^; PH3^+^ among total GFP^+^ cells. n = 800–1000 cells from 3 different brains. **P < 0.01, ns = no significant difference; one-way ANOVA followed by Bonferroni post-hoc test. Data are presented as the mean ± SEM. **(J)** E13.5 embryos were electroporated with scramble shRNA or Kif2a shRNA1, and E15.5 brains with a 24 h Brdu labeling were co-immunostained for GFP (green), Brdu (red) and Ki67 (blue). Scale bars = 50 μm. **(K)** Quantification of the percentage of GFP^+^; Brdu^+^; Ki67^-^ among total GFP^+^ cells in the VZ and SVZ. n = 600–800 cells from 3 different brains *P < 0.05; Student’s *t*-test. Data are presented as the mean ± SEM. **(L)** Immunostaining of GFP (green) and c-caspase3 (red) of E15.5 neocortex. Scale bars = 50 μm. **(M)** Quantification of the percentage of GFP^+^; c-caspase3^+^ cells among total GFP^+^ cells. n = 4 different brains. ns = no significant difference; Student’s *t*-test. Data are presented as the mean ± SEM.

Having found that silencing Kif2a increased the accumulation of newborn cells in the IZ and CP, we next asked whether this phenotype was caused by the effect of Kif2a on the proliferation of NSCs/NPCs. To test this hypothesis, scramble shRNA or Kif2a shRNA1 was introduced into E13.5 mouse brains via in utero electroporation. Brdu (50 mg/kg) was injected intraperitoneally at E15.5, and brain samples were analyzed 2 hours after Brdu injection (Fig 3A). Consistent with our findings, knocking down Kif2a significantly decreased the percentage of GFP^+^; Brdu^+^ cells among total GFP^+^ cells. Rescue experiments using Kif2a^Res^ plasmids in Kif2a-deficient cells showed that Brdu incorporation was similar to that of controls (Fig 3B). We next asked whether Kif2a played a role in regulating the proliferation of NSCs/NPCs in vitro. As shown in Fig 3C, E13.5 embryonic neocortex was dissected and digested in trypsin for 15 minutes, and single cells were cultured in proliferation medium containing B27 supplement, glutamine, hEGF (20 ng/ml) and b-FGF (20 ng/ml). Immunostaining for nestin confirmed that almost all the cells were NSCs. These NSCs were then infected with lentivirus encoding scramble shRNA or Kif2a shRNA, as indicated, with GFP signal indicating the transfected cells (Fig 3D). At 3 days after infection, knockdown of Kif2a significantly decreased the size of neurospheres compared to those derived from control cells (Fig 3E), implying an effect of Kif2a on cell proliferation. To further test this issue, NSCs/NPCs were planted onto coverslips that coated with poly-L-ornithine and laminin to maintain NSCs/NPCs in a monolayer. These cells were then incubated with the proliferation medium containing 5 μM Brdu for 6 hours (Fig 3F). Quantitative analysis showed that the proportion of GFP^+^; Brdu^+^ cells among total GFP^+^ cells was reduced by 15% compared with that in controls (Fig 3G), indicating that Brdu incorporation was decreased in Kif2a-deficient NSCs in vitro.

To further explore the effect of Kif2a on cell proliferation, we introduced scramble shRNA or Kif2a shRNA1 into E13.5 mouse neocortex via in utero electroporation. 2 days later, E15.5 slices were immunostained with phosphorylated histone H3 (PH3) antibody, a mitotic marker, to analyze the mitotic index (Fig 3H). Quantitative analysis showed that the proportion of GFP^+^; PH3^+^ cells among total GFP^+^ cells in the VZ and SVZ was significantly decreased in Kif2a shRNA1-expressing brains compared to that of controls (Fig 3I). Further, Kif2a^Res^ plasmids rescued the mitotic defects to a normal level (Fig 3H and 3I), suggesting that knocking down Kif2a decreased the number of NSCs/NPCs in mitosis. In addition, we analyzed the cell cycle exit of neocortical NSCs/NPCs. Embryos were electroporated with scramble shRNA or Kif2a shRNA1 at E13.5, and a 24 h Brdu (50mg/kg) labeling was administered at E14.5. We then co-immunostained GFP, Brdu and Ki67 to reveal the transfected cells that withdrew from the cell cycle (Brdu^+^ and Ki67^-^) in the VZ and SVZ at E15.5 (Fig 3J). The percentage of GFP^+^; Brdu^+^; Ki67^-^ cells among total GFP^+^ cells was increased after knocking down Kif2a, suggesting that more GFP^+^ NSCs/NPCs had exited the cell cycle when Kif2a was depleted (Fig 3K). We also excluded the possibility that apoptosis was responsible for the decrease of NSCs/NPCs proliferation, as the immunostaining of cleaved caspase-3 (c-caspase3) was not significantly different between the two groups (Fig 3L and 3M). Taken together, these results demonstrated that knocking down Kif2a in E13.5 neocortical NSCs/NPCs inhibited their proliferation in vivo and in vitro.

### Kif2a regulates the switch of NSCs/NPCs from proliferation to differentiation

**Fig 4:**
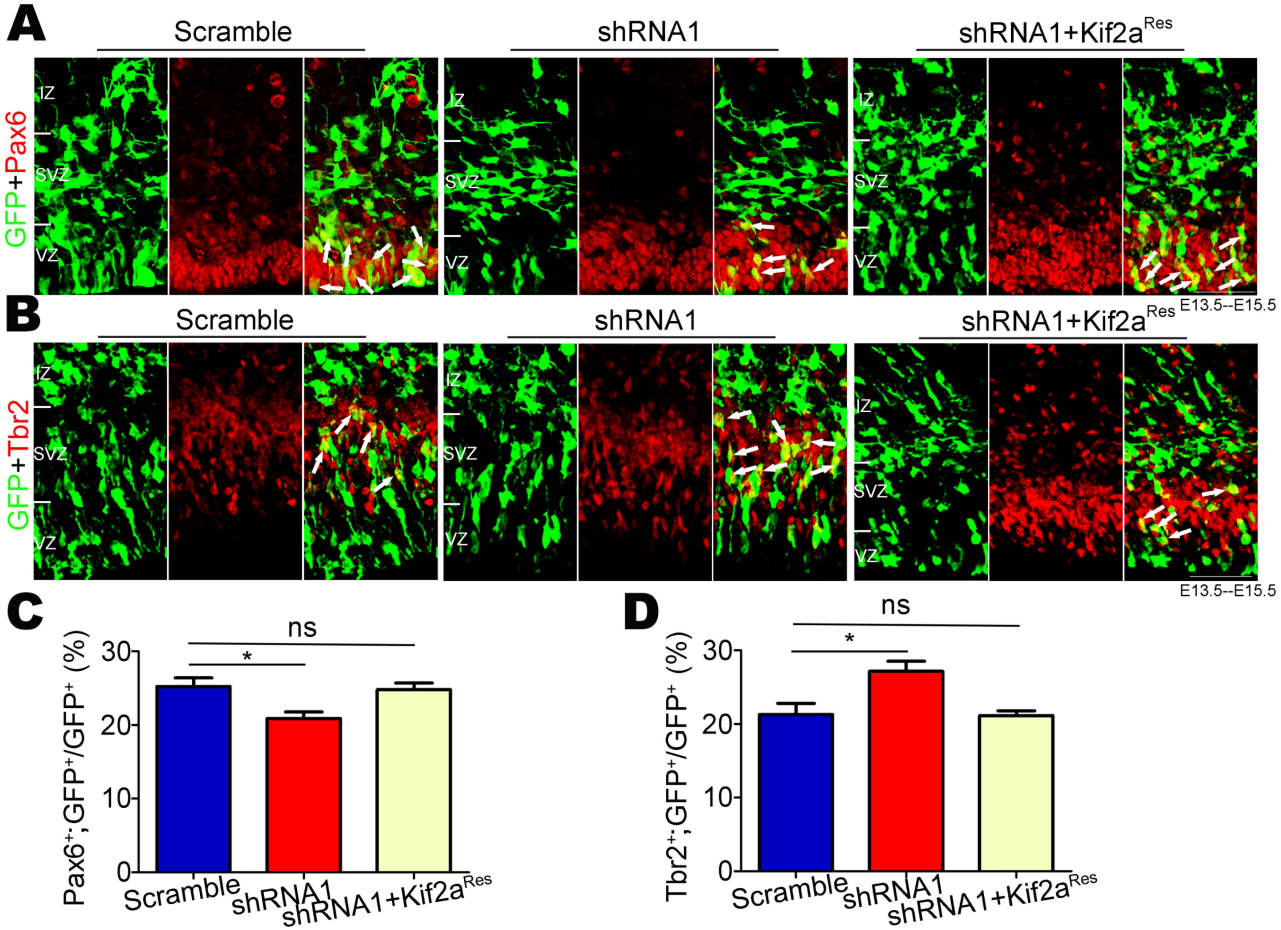
Kif2a regulates the switch of NSCs/NPCs from proliferation to differentiation. **(A-B)** Indicated plasmids (scramble shRNA, Kif2a shRNA1 or Kif2a shRNA1+Kif2a^Res^) were introduced into E13.5 neocortex via in utero electroporation, and electroporated brain slices were co-immunostained for GFP (green) with Pax6 (red) or Tbr2 (red) at E15.5. Scale bars = 50 μm. **(C-D)** Quantification of the percentages of GFP^+^;Pax6^+^ or GFP^+^;Tbr2^+^ among total GFP^+^ cells. n = 300–500 cells from three different brains. *P < 0.05, **P < 0.01, ns = no significant difference; one-way ANOVA followed by Bonferroni post-hoc test. Data are presented as the mean ± SEM.

Increased accumulation of newborn cells in the IZ and CP may result from increased differentiation of NSCs/NPCs. To explore this hypothesis, scramble shRNA and Kif2a shRNA1 constructs were transfected separately into the E13.5 neocortex via in utero electroporation. RGCs and IPCs were immunostained using anti-Pax6 and anti-Tbr2 antibodies, respectively (Fig 4A and 4B). Interestingly, compared with control brains, Kif2a shRNA1-expressing brains showed more GFP^+^ cells that migrated into Tbr2^+^ layers but fewer GFP^+^ cells that remained in Pax6^+^ layers. Quantitative analysis also revealed a decrease in the proportion of GFP^+^;Pax6^+^ cells among total GFP^+^ cells but an increase in the proportion of GFP^+^;Tbr2^+^ cells among total GFP^+^ cells after silencing Kif2a (Fig 4C and 4D). Moreover, we found that Kif2a^Res^ plasmids rescued the abnormal phenotype caused by decreasing the expression level of Kif2a (Fig 4C and 4D).

### Kif2a-deficiency promotes the differentiation of NSCs/NPCs in vivo and in vitro

**Fig 5:**
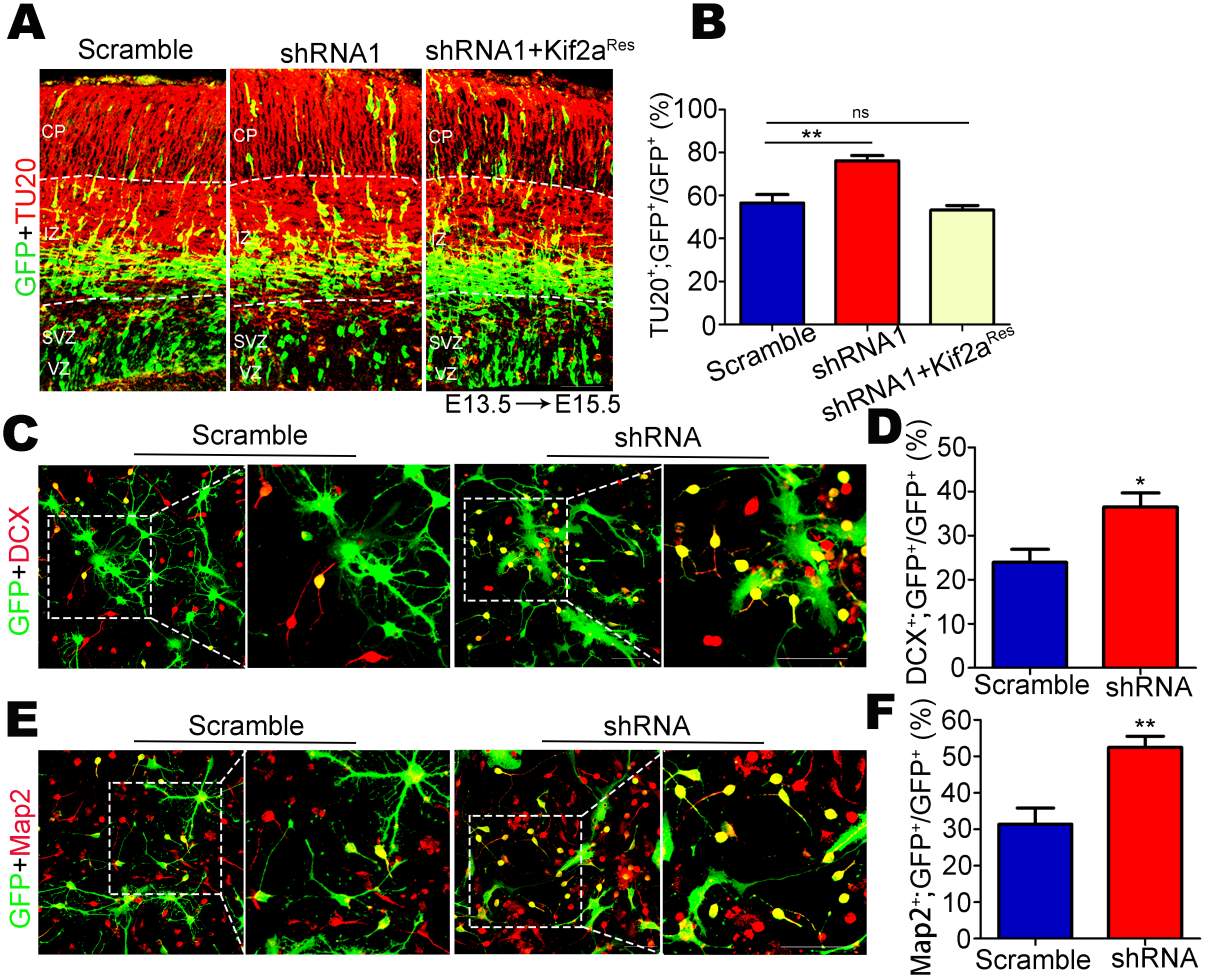
Kif2a is involved in the differentiation of NSCs/NPCs. **(A)** Mouse embryos were electroporated with indicated plasmids (scramble shRNA, Kif2a shRNA1 or Kif2a shRNA1+Kif2a^Res^) at E13.5, and analyzed at E15.5. GFP (green) represents cells expressing the indicated plasmids; TU20 (red) represents immature neurons. Scale bars = 50 μm. **(B)** Quantitative analysis of the percentage of GFP^+^;TU20^+^ cells among the total GFP^+^ cells showed that more GFP-labeled TU20^+^ neurons were located in IZ and CP layers after knockdown of Kif2a. n = 800–1000 cells from three different brains. **P < 0.01, ns = no significant difference; one-way ANOVA followed by Bonferroni post-hoc test. Data are presented as the mean ± SEM. **(C, E)** Cultured NSCs/NPCs transfected with lentivirus expressing scramble shRNA or Kif2a shRNA were incubated in differentiation medium for 3 days. Co-immunostaining for GFP (green) with the neuronal marker DCX (red) in (C) or Map2 (red) in (E). Scale bars = 50 μm. **(D, F)** Quantification of the percentage of GFP^+^;DCX^+^ or GFP^+^;Map2^+^ cells among total GFP^+^ cells showed that knocking down Kif2a increased the number of newborn neurons. n = 600–800 cells from three different experiments. *P < 0.05; **P < 0.01; Student’s *t*-test. Data are presented as the mean ± SEM.

To address whether knocking down Kif2a promotes the differentiation of NSCs/NPCs, we electroporated E13.5 embryos with scramble shRNA or Kif2a shRNA1 plasmids. At E15.5, we examined newborn neurons by using TU20 antibody to recognize β-III tubulin (neuronal tubulin), a marker for immature neurons, and analyzed the percentage of GFP^+^; TU20^+^ cells among total GFP^+^ cells (Fig 5A). Consistent with the altered localization of GFP^+^ cells in the neocortex, the Kif2a shRNA1-expressing group also showed a significantly higher proportion of immature neurons in the IZ and CP. Importantly, expression of Kif2a^Res^ plasmids restored the proportion of immature neurons in these regions to that in control brains (Fig 5B).

We next investigated whether Kif2a regulates the differentiation of NSCs/NPCs in vitro. To that end, NSCs/NPCs transfected with scramble shRNA or Kif2a shRNA were plated onto coverslips coated with poly-L-ornithine and laminin and were cultured for 3 days with differentiation medium in the absence of hEGF and bFGF. Cells were then immunostained for the neural markers such as Doublecortin (DCX) and Microtubule-associated protein 2 (Map2) to assess neurogenesis (Fig 5C and 5E). Consistent with previous results, the percentages of GFP^+^;DCX^+^ and GFP^+^;Map2^+^ cells among the total GFP^+^ cells were significantly increased after knockdown of Kif2a (Fig 5D and 5F). These results thus suggested that Kif2a deficiency promoted the differentiation of NSCs/NPCs in vivo and in vitro.

### The level of β-catenin in cortical NSCs/NPCs is regulated by Kif2a

**Fig 6:**
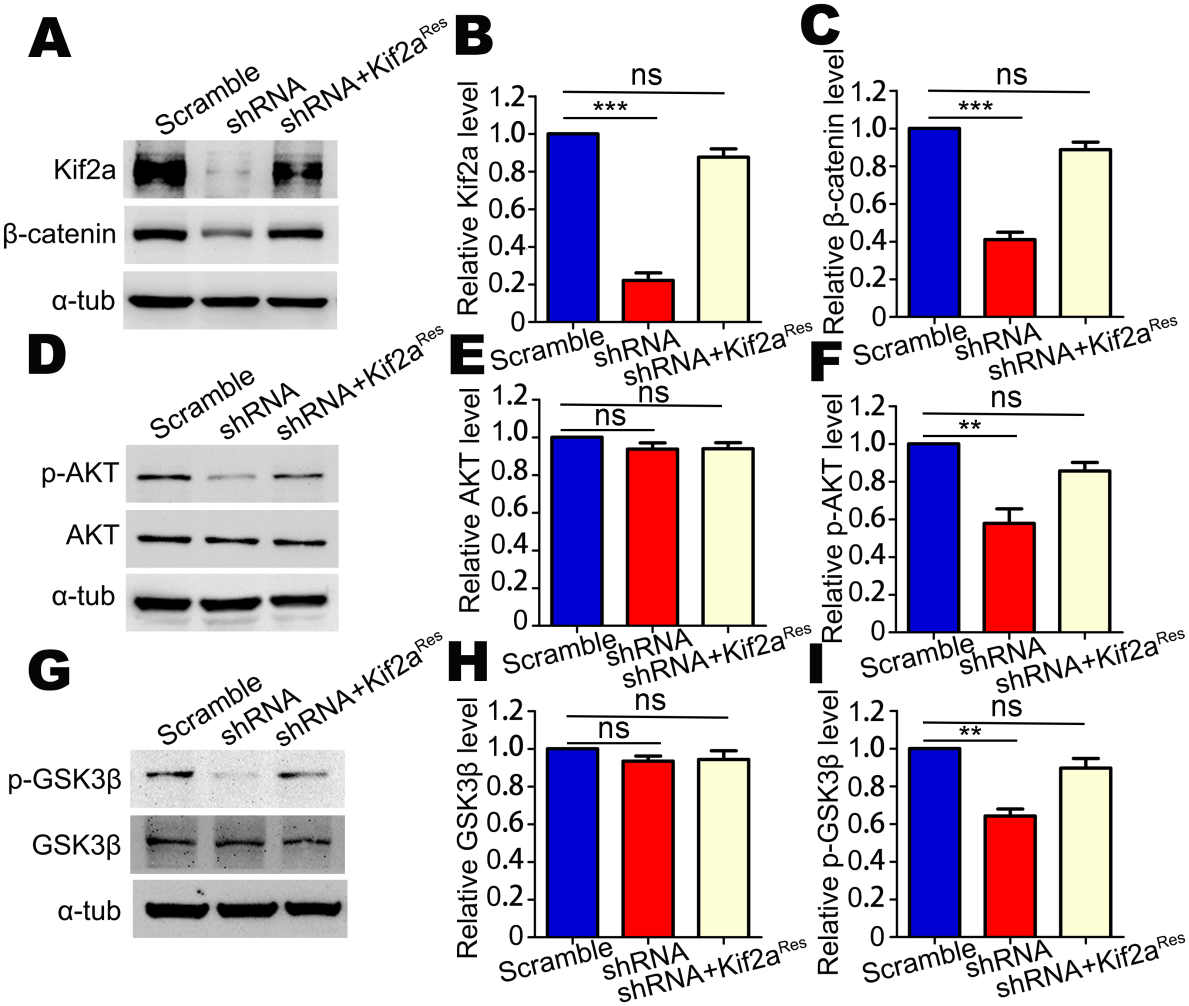
The expression of β-catenin, GSK3β, p-GSK3β, AKT, and p-AKT in NSCs/NPCs after Kif2a knockdown. **(A)** Western blot analysis of the Kif2a and β-catenin levels in lysates of NSCs transfected with indicated plasmids (scramble shRNA, Kif2a shRNA or Kif2a shRNA+Kif2a^Res^). **(B-C)** Quantitative analysis of the Western blot data in A. n = 3 per group. ***P < 0.001; ns = no significant difference; one-way ANOVA followed by Bonferroni post-hoc test. Data are presented as the mean ± SEM. **(D)** Western blot analysis of the AKT and p-AKT levels in lysates of NSCs transfected with indicated plasmids (scramble shRNA, Kif2a shRNA or Kif2a shRNA+Kif2a^Res^). **(E-F)** Quantitative analysis of the Western blot data in D. n = 3 per group. **P < 0.01; ns = no significant difference; one-way ANOVA followed by Bonferroni post-hoc test. Data are presented as the mean ± SEM. **(G)** Western blot analysis of the GSK3β and p-GSK3β levels in lysates of NSCs transfected with indicated plasmids (scramble shRNA, Kif2a shRNA or Kif2a shRNA+Kif2a^Res^). **(H-I)** Quantitative analysis of the Western blot data in G. n = 3 for each. **P < 0.01; ns = no significant difference; one-way ANOVA followed by Bonferroni post-hoc test. Data are presented as the mean ± SEM.

β-Catenin is an essential component of canonical Wnt signaling, which has been implicated in regulating the balance between proliferation and differentiation of NSCs/NPCs [29]. Previous studies showed that conditional deletion of β-catenin from neocortical NSCs/NPCs resulted in increased numbers of intermediate progenitors [30]. Thus, manipulations of Kif2a and β-catenin produce similar effects on neocortical neurogenesis. To explore this relationship, cultured NSCs were infected with lentivirus expressing scramble shRNA or Kif2a shRNA. Surprisingly, we found that the level of β-catenin was significantly decreased after knockdown of Kif2a (Fig 6A, 6B and 6C). In addition, expression of Kif2a^Res^ plasmids restored the β-catenin to a normal level (Fig 6C). It is known that β-catenin acts downstream of the PI3K-AKT-GSK3β signaling pathway and is regulated by GSK3β through the ubiquitin-dependent proteasome pathway [31]. We detected the levels and activities of AKT and GSK3β via Western blot (Fig 6D and 6G). Knockdown of Kif2a had no effect on total AKT (Fig 6E) and total GSK3β (Fig 6H) but significantly decreased the phosphorylation of AKT (p-AKT) (Fig 6F) and GSK3β (p-GSK3β) (Fig 6I), indicating that the activity of GSK3β was increased in Kif2a shRNA-expressing cells compared with that in control cells. Furthermore, expression of Kif2a^Res^ plasmids rescued the decrease of p-AKT and p-GSK3β caused by knocking down Kif2a (Fig 6F and 6I). Taken together, these findings support a model that Kif2a regulates neocortical neurogenesis in a manner similar to that of β-catenin.

## Discussion

During embryonic neocortical development, the activities of NSCs/NPCs are precise and complex, requiring proper control of self-renewal, cell fate specification, differentiation and survival. Any disruption of these processes may impair the balance and lead to neuronal disorders. However, the mechanisms regulating these processes are not yet fully understood.

Here, we provide evidence for Kif2a’s functions in regulating embryonic neocortical neurogenesis. We found that Kif2a was highly expressed in NSCs/NPCs as well as neurons from E13.5 neocortex, but was weakly detected in the VZ/SVZ at E15.5 and E17.5, which suggested a potential role of Kif2a in NSCs/NPCs at the peak stage of embryonic neocortical neurogenesis (E13.5) (Fig 1). We found that Kif2a regulated the neocortical localization of newborn cells. Knocking down Kif2a decreased the newborn cells that were present in the VZ/SVZ, whereas increased the accumulation of newborn cells in the IZ and CP. In light of the phenotype mentioned above, there are four reasons may contribute to the abnormal distribution of Kif2a-deficient newborn cells. First, we explored whether Kif2a regulated cell migration in the neocortex. As shown in Fig 2H and 2I, during the critical period of neuronal migration, knocking down Kif2a in E15.5 NSCs/NPCs did not alter the localization of newborn cells analyzed at E18.5, demonstrating Kif2a’s little effect on cell migration in the neocortex. However, a migratory defect of cortical neurons was observed in *Kif2a*^*-/-*^ mice compared to wild type (WT) mice [18], and Kif2a regulated HeLa cell migration [22]. Notice that neuronal migration was regulated by multiple intrinsic and extrinsic factors in vivo, and Kif2a was knocked out throughout the brain, It is thus of considerable interest to investigate whether Kif2a indirectly delayed neuronal migration in *Kif2a*^*-/-*^ mice. Second, we explored whether Kif2a regulated the proliferation of neocortical NSCs/NPCs. As is reported, Kif2a regulates bipolar spindle assembly during mitosis in cultured human U2OS cells [24], and Kif2a depletion generates chromosome segregation defects in animal cap cells [25]. In addition, Kif2a drives primary cilia disassembly coupled with cell proliferation via its microtubule-depolymerizing activity in hTERT-RPE1 cells [23]. Although these reports demonstrate Kif2a’s functions in mitosis and proliferation in cultured cells, it remains unclear about Kif2a’s roles in embryonic neocortical NSCs/NPCs. In this study, we found that knocking down Kif2a in neocortical NSCs/NPCs significantly decreased their proliferation in vivo and vitro (Fig 3), which may explain the reduction of GFP^+^ cells resided in the VZ/SVZ. Third, apoptosis of NSCs/NPCs in the VZ and SVZ may also contribute to the reduction of GFP^+^ cells resided in the VZ/SVZ layers. However, no significant difference of c-caspase3 positive cells was found in control and Kif2a shRNA1-expressing brains (Fig 3L and 3M), we thus excluded the possibility that knocking down Kif2a increased cell apoptosis. Fourth, knocking down Kif2a enhances neocortical neurogenesis by increasing cell cycle exit, and promoting neuronal differentiation. This is based on our observations that knocking down Kif2a elevated the percentage of GFP^+^;Brdu^+^;Ki67^-^ cells among total GFP^+^ cells (Fig 3J and 3K), as well as increased the number of newborn neurons in vivo and in vitro (Figs 4 and 5). Taken together, Kif2a plays a critical role in embryonic neocortical neurogenesis, including promotes NSCs/NPCs proliferation or self-renewal and suppresses neuronal differentiation.

As we know, MT dynamics has long been implicated in various aspects of neuronal development as well as in neurogenesis. Recent studies have demonstrated that many genes are involved in regulating the proliferation and differentiation of NSCs/NPCs. Among these genes, Hook3, DOCK7, Axin, Smek1 and some known microRNAs have all been shown to play important roles in regulating MT activities and also to be involved in neurogenesis [32–36]. However, how does Kif2a, as a MT-depolymerizing protein, regulate embryonic neocortical neurogenesis? In this study, we found that Kif2a regulated β-catenin level by affecting the activity of GSK3β, and Kif2a-deficient NSCs showed lower β-catenin level than that of control cells. We are aware of the report that inhibition of β-catenin signaling during embryonic development causes NSCs to prematurely exit the cell cycle and then differentiate into neurons [8]. It is thus of great significance to further investigate the mechanism that Kif2a regulates β-catenin level. Recent study has shown that silencing Kif2a in Tca8113 cells inhibits the PI3K/AKT signaling pathway [37], which is consistent with our findings of that in Kif2a-deficient NSCs. It is also reported that the MT depolymerization activity of Kif2a was regulated by CDK5, PAK1 and ROCK2 kinases during neuronal development [38]. However, whether PI3K/AKT signaling pathway in turn regulates the activity of Kif2a remains largely unknown, which is worth of future studies.

## Conclusion

In summary, we propose that Kif2a is highly expressed in NSCs/NPCs as well as neurons at the early stage of embryonic development and is involved in regulating neocortical neurogenesis.
